# The Impact of Progressive Pulmonary Fibrosis in Systemic Sclerosis–Associated Interstitial Lung Disease

**DOI:** 10.3390/jcm12206680

**Published:** 2023-10-23

**Authors:** María Martín-López, Patricia E. Carreira

**Affiliations:** 1 Department of Rheumatology, Hospital Universitario 12 de Octubre, 28041 Madrid, Spain; marymartin12@hotmail.com; 2 Instituto de Investigación Hospital 12 de Octubre (imas12), 28041 Madrid, Spain

**Keywords:** systemic sclerosis, interstitial lung disease, progressive pulmonary fibrosis, biological therapies, antifibrotics

## Abstract

Systemic sclerosis (SSc) is an autoimmune connective tissue disease characterized by immune dysregulation and progressive fibrosis, typically affecting the skin, with variable internal organ involvement. Interstitial lung disease (ILD), with a prevalence between 35 and 75%, is the leading cause of death in patients with SSc, indicating that all newly diagnosed patients should be screened for this complication. Some patients with SSc-ILD experience a progressive phenotype, which is characterized by worsening fibrosis on high-resolution computed tomography (HRCT), a decline in lung function, and premature mortality. To assess progression and guide therapeutic decisions, regular monitoring is essential and should include pulmonary function testing (PFT), symptom assessment, and repeat HRCT imaging when indicated. Multidisciplinary discussion allows a comprehensive evaluation of the available information and its consequences for management. There has been a shift in the approach to managing SSc-ILD, which includes the addition of targeted biologic and antifibrotic therapies to standard immunosuppressive therapy (particularly mycophenolate mofetil or cyclophosphamide), with autologous hematopoietic stem-cell transplantation and lung transplantation reserved for refractory cases.

## 1. Introduction

Systemic sclerosis (SSc) is a rare multiorgan inflammatory disease characterized by dysregulated fibrosis affecting skin and internal organs, microvascular damage, and phenotype-specific serum autoantibodies. Interstitial lung disease (ILD) is frequent in SSc patients, with a prevalence ranging from 35% to 75%, and is associated with increased morbidity and mortality [1,2]. Given the high prevalence and poor prognosis of ILD, it is recommended that every SSc patient be screened for ILD at diagnosis, even if respiratory symptoms are absent [3]. In addition to assessment of clinical symptoms and auscultation of the chest to detect the typical “velcro” crackles, ILD screening should include high-resolution computed tomography (HRCT) and pulmonary function testing (PFT).

The presentation of SSc-ILD in SSc patients is not homogeneous in terms of both severity and outcome. While some patients may present mild and stable ILD for many years, others may develop severe and rapidly progressive lung dysfunction. To avoid delayed diagnosis of the most severe forms of SSc-ILD, close monitoring of all SSc patients is an essential part of disease management. Overall, 20–30% of patients with SSc-ILD will develop progressive pulmonary fibrosis (PPF) over time, while roughly 50% of patients will remain stable, and some may even demonstrate improved lung function later in the disease course [4].

However, predicting which patients with SSc-ILD will develop PPF remains a challenge. Various studies have identified several predictors of SSc-ILD progression (i.e., male sex, diffuse cutaneous disease), but translating these observations into clinical practice is difficult, in part because of the different definitions of ILD progression considered in each study (i.e., different thresholds of forced vital capacity (FVC) and diffusing capacity for carbon monoxide (DLCO) decline).

The management of progressive fibrosing (PF)-ILD is challenging for clinicians, primarily because of the insufficient availability of high-quality data concerning the efficacy and safety of specific therapies in this condition. To address this, some experts have suggested that idiopathic pulmonary fibrosis (IPF) should be grouped with PPF-ILD, as they share similar pathogenic mechanisms and prognosis [5]. Currently, only two antifibrotic agents, nintedanib and pirfenidone, have been approved worldwide as effective treatments for slowing the decline of lung function in IPF patients.

Research in SSc-ILD on this topic may help clinicians identify patients who need closer monitoring while receiving standard immunomodulatory therapy. In addition to cyclophosphamide (CYC), randomized controlled trials (RCTs) over the past decade have demonstrated some degree of efficacy of four therapeutic agents in SSc-ILD: mycophenolate mofetil (MMF), nintedanib, rituximab (RTX), and tocilizumab (TCZ) [6,7,8,9]. The expansion of the treatment armamentarium represents a major advance for this rare disease and offers the opportunity to personalize the management of patients with SSc-ILD.

In this report, we present a critical review of the published literature on the novel concept of PPF, the significance of detecting patients with PF SSc-ILD and its associated risk factors, and the available evidence to guide the treatment of patients with PF SSc-ILD.

## 2. Identification of Patients with Progressive Fibrosing SSc-ILD and Diagnosis

A percentage of patients with connective tissue disease (CTD)-ILD develop a PF phenotype, the main features of which are an increase in fibrotic changes in the lung (i.e., traction bronchiectasis and honeycombing) on HRCT, worsening PFT, worsening symptoms, and increased mortality [1,4]. Because of the implications for patient counseling and management, it is important to identify the progression of fibrosing ILD as early as possible. In SSc, the variable rates of disease progression and response to treatment emphasize the necessity for close monitoring of all SSc-ILD patients following diagnosis and initiation of treatment [10,11,12].

Many patients with severe SSc-ILD develop this complication within the first 5 years of disease, although some SSc-ILD patients may remain stable for some time and show progression later in the disease course [13]. In an attempt to identify the different outcomes from different progression patterns and the risk factors associated with the development of PF-ILD, Hoffmann-Vold et al. recently analyzed the presence of progression in 826 patients with SSc-ILD and long-term follow-up, included in the European Scleroderma Trials and Research (EUSTAR) database. Over the course of 12 ± 3 months, 219 (27%) patients exhibited PF ILD with either moderate (FVC decline ranging between 5% and 10%) or significant (FVC decline greater than 10%). In any 12-month time frame, 23% to 27% of patients with SSc-ILD developed PF ILD, although only a minority had a progression in consecutive periods. The most common pattern in patients with progressive ILD (58%) was characterized by a gradual decline in lung function and more frequent periods of stability/improvement compared to decline. On the other hand, about 8% showed the opposite pattern, with a rapid, continuous decline in FVC [4]. Similarly, recent observational studies have shown that approximately one-third of patients with SSc-ILD experience PPF. In a national Norwegian cohort including 391 patients with SSc-ILD and a mean follow-up period of 6 years, 33% presented with severe progression of ILD, defined as a decline in FVC > 10% of predicted or a decline in FVC between 5 and 10% of predicted, with a decline in DLCO ≥ 15% of predicted [1].

There are no published clinical practice guidelines for the detection and early diagnosis of ILD-SSc or for monitoring its progression [14]. The British Society for Rheumatology (BSR)/British Health Professionals in Rheumatology (BHPR) guideline for the management of SSc recommends that all patients with SSc should be assessed for pulmonary fibrosis but does not describe methods of screening or monitoring [15].

Given the significance of timely recognition and management of ILD, it is crucial to prioritize screening and early diagnosis. It should be noted that SSc-ILD might be present even if there are no respiratory symptoms or a restrictive defect [1,16]. It is well known that PFT has very limited sensitivity for the diagnosis of ILD and may be influenced by extra-pulmonary factors that may be present in SSc, such as fatigue, microstomia, severe skin involvement, or myopathy. Nevertheless, PFT remains essential in monitoring the progression of ILD-SSc [17]. HRCT has been demonstrated to be superior to PFT for the detection of SSc-ILD in the early stages [16] and is regarded as the gold standard for diagnosing ILD. A recent Delphi expert consensus study stated that every SSc patient should be screened for ILD at diagnosis [10]. Screening includes symptom assessment, chest auscultation, PFT, and HRCT. Other factors contributing to progressive symptoms, such as heart involvement or pulmonary arterial hypertension (PAH), should also be taken into account given the complex nature of lung-associated manifestations of SSc. Clinical symptoms and chest auscultation should be assessed during follow-up throughout the course of the disease, and PFT should be repeated periodically. Protocols for monitoring SSc-ILD patients vary, but there is consensus that PFT (FVC and DLCO) and respiratory symptoms should be evaluated at least every 6 months for the first 3 to 5 years after the onset of the first non-Raynaud’s symptom to adequately monitor for possible progression of ILD [11,13,18]. However, in SSc patients without ILD or with controlled ILD after the first 3–5 years, annual PFT is considered sufficient to monitor both the onset and progression of SSc-ILD and to detect SSc-associated PAH [12]. The frequency of repeat HRCT scans is not well defined in the literature. Most experts would recommend that the decision to perform a repeat HRCT be based on worsening PFT and worsening or new symptoms such as dyspnea and cough, taking into account the presence of risk factors such as male sex or anti-topoisomerase I antibodies (ATA) [10,11].

Specific laboratory tests may indicate the presence or progression of ILD, including elevated circulating C-reactive protein (CRP) and other acute-phase reactants. Like CRP, elevated serum levels of interleukin (IL)-6 were associated with lung function decline in the first year and with death during the first 30 months of follow-up in an early cohort of SSc-ILD patients [19]. Although it is possible that IL-6 will play a role in lung fibrosis, both IL-6 and CRP are non-specific for ILD, as their levels can be elevated in almost any inflammatory condition.

Several biomarkers currently under investigation have shown utility in the detection and monitoring of ILD in various diseases. Krebs von den Lungen-6 (KL-6) is a glycoprotein expressed mainly by type II pneumocytes, especially in those cells in the process of proliferation and regeneration. C chemokine ligand 18 (CCL-18), formerly known as lung activation chemokine, is constitutively expressed by lung tissue macrophages and dendritic cells, and it is highly inducible by inflammatory stimulus. It is considered an indicator of lung fibrotic remodeling since it can induce collagen synthesis by lung fibroblasts, thus contributing to lung function deterioration. Serum levels of both proteins have been correlated with the severity of ILD and have been associated with a worse course of this complication in SSc patients [20]. Similarly, immunosuppressive treatment has been shown to reduce their levels in parallel with the improvement or stabilization of lung function observed in these patients [21]. Lung epithelial–derived surfactant protein D (SP-D) exerts its function as a component of the innate immune response and plays a role in immune and inflammatory regulation within the lung. In a large European study, including 427 SSc patients, SP-D levels at baseline strongly predicted the presence of ILD but were not associated with the worsening of ILD in longitudinal follow-up [20]. Despite all these studies, relatively little research has been carried out in this area, and more data in larger longitudinal cohorts is needed to better identify patients at risk for ILD-SSc development and progression.

## 3. Definition of PPF

Of paramount importance in identifying ILD progression and characterizing clinical phenotypes of PF SSc-ILD is reaching a consensus on how to define progression, given the various definitions that have been proposed and are currently available [22,23,24]. All of these definitions are multidimensional, including symptoms, functional assessments, and imaging to better capture clinically relevant progression.

Cottin et al., 2018 proposed the following criteria for the definition of PF-ILD: a relative decrease in FVC ≥ 10%, a relative decrease in the DLCO ≥ 15%, or a relative decrease in FVC ≥ 5% but <10% combined with worsening of symptoms or radiographic findings in the previous 24 months [25]. OMERACT (Outcome Measures in Rheumatology) has proposed the definition of “clinically meaningful progression” of CTD-ILD based on the PFT parameters [26]; this definition can be applied to SSc-ILD, and it is the same as the “ILD progression” proposed by Goh et al. in 2017 [22]. OMERACT defines progression as a relative decline in FVC ≥ 10%, or a relative decline in FVC ≥ 5% but <10%, associated with a relative decline in the DLCO ≥ 15% over 12 months [26], which have been identified as surrogate biomarkers for mortality [4,13,23,24,25,27].

In contrast, the eligibility criteria proposed for the INBUILD study (Flaherty et al., 2019), which included ILD of various etiologies other than IPF, all with a progressive pattern, were: a relative decline in FVC ≥ 10%; a relative decline in FVC ≥ 5% but <10% combined with worsening of respiratory symptoms or increased extent of fibrosis on HRCT; or worsening of respiratory symptoms combined with increased extent of fibrosis on HRCT, all within the previous 24 months [24].

Recently, the American Thoracic Society/European Respiratory Society (ATS/ERS) formed a joint committee with the Japanese Respiratory Society (JRS) and the Asociacion Latinoamericana de Torax (ALAT) and proposed updates on the diagnosis and management of IPF and other types of PPF. In this guideline, PPF was defined based on three categories: worsening of respiratory symptoms; evidence of disease progression by functional assessment as reflected by an absolute decline in FVC > 5% predicted or an absolute decline in DLCO > 10% predicted; and radiologic evidence of disease progression [23]. In this description, ILD progression is considered to be not only the extent of ILD on HRCT but also the appearance of new areas of ILD or the transition from one ILD pattern to another. The definition ensures that ILD is relevant through the simultaneous occurrence of worsening symptoms and HRCT progression. The PPF concept can be applied to fibrotic ILD patients of known or unknown etiology other than IPF. Compared to OMERACT [26] and Goh et al. [22], the consensus definition [23] has defined “functional progression” as an absolute decline in FVC ≥ 5% or an absolute decline in DLCO corrected ≥10% over 12 months. This new definition of PPF needs to be validated and compared to the previous ones, particularly with regard to its prognostic impact in SSc-ILD. The main definitions of PPF for each of the proposed criteria and the underlying study are described in Table 1.

## 4. Risk Factors for Progressive SSc-ILD

Several studies have attempted to elucidate the most important factors that may predict ILD progression in SSc. Although some factors (i.e., male sex) have been consistently found to predict outcomes in SSc-ILD [4,27,29], other factors have shown inconsistent predictive potential, such as African American race [30,31] and plasma CCL18 and CXCL4 levels [32,33]. The predictive value of these biomarkers in observational studies and RCTs varies depending on the population studied and on how SSc-ILD progression is defined.

As mentioned above, male sex is independently associated with a higher risk of developing ILD. In a large cohort of 2686 consecutive new SSc patients reported by Peoples et al., males were more likely to have diffuse cutaneous SSc (dcSSc) and ILD, with a significantly reduced survival rate [27]. In addition, male patients with SSc were significantly more likely to have ever been cigarette smokers and to have environmental exposures. Recently, an analysis of the EUSTAR database was published to determine the impact of gender on outcomes in patients with SSc-ILD. A total of 1136 male and 5253 female patients with SSc-ILD were included. Disease duration and the percentage of predicted FVC in males were associated with greater disease progression. In the survival analysis, male sex was a predictor of mortality [34].

Patients with an older age at the onset of SSc represent an at-risk subgroup and should be evaluated more frequently for potential organ involvement. In a large German registry of 3281 SSc patients, older dcSSc patients developed lung fibrosis significantly more often (73.5%, *p* < 0.001) compared to the younger cohort (55.3%) [35]. Other demographic factors, such as race, have also been analyzed, and the results showed that ILD is more severe in African Americans compared to European ancestry in SSc patients [30].

However, the major risk factors for progression of SSc-ILD include diffuse involvement of the skin, the presence of ATA, elevated CRP, worse PFT at baseline, and greater ILD extent on HRCT [13,36]. Pulmonary physiology is the best-studied marker of disease progression. Restrictive PFT and impaired gas exchange have consistently been shown to be independent predictors of poor outcomes [22,37,38,39]. Dynamic monitoring of PFT over time is useful to better predict the course of SSc-ILD. A decrease in FVC > 10%, or of 5–9% with an associated 15% decrease in DLCO, identifies a population at particularly high risk of death [22]. However, other disease-related factors, such as worsening myopathy, fatigue, or increased skin thickness, should also be taken into account when assessing a decrease in PFT in SSc patients. Cross-sectional imaging has also been the subject of extensive research as a prognostic tool. The initial observation by Goh et al. [22], that fibrosis involving more than 20% of the lung parenchyma is associated with a significant increase in the odds of mortality, has been confirmed in several independent cohorts [38,40]. The presence of a usual interstitial pneumonia (UIP) pattern on imaging or lung pathology is also associated with an aggressive form of ILD [40].

In addition, other SSc organ manifestations, such as gastroesophageal reflux disease [41], arthritis, cardiac or renal involvement, digital ulcers, and shorter disease duration, have been associated with the presence of ILD [36,38,39,42]. In IPF, for example, treatment of gastroesophageal reflux may slow progression [43]. In SSc, a lack of esophageal contractility is associated with more severe restrictions on PFT [42]. These findings suggest that the initial assessment of reflux symptoms in SSc-ILD may help to stratify the individual patient risk. Further research is needed to determine how to optimize the treatment of reflux disease in the hope that such treatment may reduce the risk or severity of PPF.

Other biomarkers, such as KL-6, CXCL4, CCL2, CCL18, or surfactant protein-D (SP-D), may predict the progression of SSc-ILD but are not available in clinical practice and are currently used almost exclusively in exploratory clinical research [13,32,44].

Biomolecular profiling via gene expression studies (i.e., exosomes, mitochondrial DNA, microRNA, transcriptomics) may help to identify patients at higher risk of disease progression based on studies developed in cohorts of patients with established SSc. Immune dysregulation, such as upregulated expression of HLA-DRB5, has been reported in patients with ILD-SSc compared with those without ILD [45]. Telomere shortening also correlates with a poorer outcome in chronic fibrosing ILDs with a PF phenotype, including ILD-SSc [46]. Furthermore, few studies have investigated epigenetic factors in SSc-ILD patients, including CpG methylation, which is associated with increased DNA methyltransferase expression in fibroblasts [47].

All of these findings have important clinical implications for the management of SSc-ILD patients, as early treatment is needed for patients at high risk of progression. The approach to identifying ILD-SSc at higher risk of progression represents a paradigm shift in SSc-ILD and highlights the need for reliable and accessible predictive factors for the progression of SSc-ILD, which may help to individualize appropriate and timely follow-up of SSc-ILD patients.

**Figure 1:**
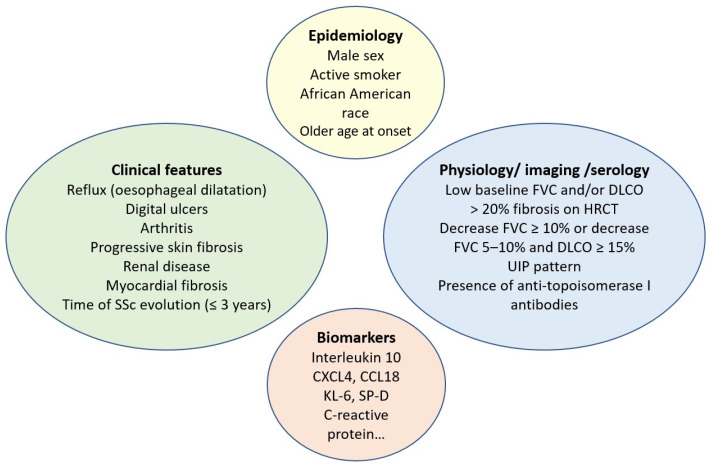
Risk factors for systemic sclerosis-associated interstitial lung disease progression. SSc: systemic sclerosis; FVC: forced vital capacity; DLCO: diffusing capacity for carbon monoxide; HRCT: high-resolution computed tomography; UIP: usual interstitial pneumonia; KL-6: Krebs von den Lungen 6; SP-D: surfactant protein-D.

In contrast to ATA, the presence of anti-centromere antibodies (ACA) is associated with a reduced risk of ILD. However, it is important to note that even patients with limited cutaneous SSc and those carrying ACA can develop ILD. It is also known from the serologic profile that anti-RNA polymerase III antibodies are associated with an intermediate risk of ILD [48]. Risk factors associated with SSc-ILD progression are detailed in Figure 1.

## 5. PPF Treatment in SSc-ILD Patients

There is no specific guideline for the management of SSc-ILD [14]. The 2016 BSR/BRHC guideline for SSc states that the decision about ILD treatment is determined by the extent, severity, and likelihood of progression. CYC is the recommended first-line treatment, but MMF is also considered an alternative or a maintenance therapy after CYC [15]. The updated EULAR 2017 recommendations for the treatment of SSc, including information published up to 2014, state that CYC, despite its known toxicity, should be considered for the management of SSc-ILD, especially for patients with PF ILD. On the other hand, the recommendations limit the use of Haematopoietic Stem Cell Transplantation (HSCT) for selected patients with rapidly PF SSc at risk of organ failure, with careful patient selection to avoid mortality [49]. The new BSR/BHPR guideline [50] as well as the updated 2023 recommendations are eagerly awaited, as they will include information on all the clinical trials performed in SSc-ILD during the last years and will include indications for both immunomodulatory and antifibrotic therapy.

In recent decades, there has been an increasing focus on SSc-ILD with the aim of achieving earlier diagnosis and improving therapeutic interventions to prevent the development of severe pulmonary fibrosis. Despite the immunosuppressive therapy currently used in most patients, SSc-ILD can progress, indicating that there is an urgent need for new therapies that can modify this poor prognosis by targeting both inflammation and fibrosis, the two main features present in SSc-ILD.

Prior to inclusion in the treatment decision algorithm for ILD-SSc, it is important to correctly classify the patient by considering several dimensions of disease severity: subsets of subclinical or clinical ILD; degree of ILD by HRCT; and functional consequences based on FVC and/or DLCO [10,11]. Immunomodulatory treatment should be considered in all patients with clinical ILD [10,15]. For patients with subclinical ILD, it will be necessary to assess the risk of progression for more accurate stratification, which could determine whether or not a particular patient is a candidate for pharmacological treatment.

Immunosuppressive therapy is the mainstay of management for SSc, although there is limited evidence of its efficacy in slowing ILD progression. The use of CYC and MMF in the treatment of SSc-ILD is supported by evidence from 2 RCTs [6,51,52,53]. The Scleroderma Lung Study I (SLS-I) and Scleroderma Lung Study II (SLS-II) are two landmark RCTs addressing the use of these drugs in patients with SSc-ILD. In SLS-I, one year of oral CYC had a significant but modest beneficial effect on dyspnea, lung function, skin involvement, and health-related quality of life in SSc-ILD patients with a disease duration of less than 7 years [52]. However, with the exception of a sustained effect on dyspnea, these effects disappeared and were no longer present at 24 months. In addition, CYC treatment was not free of adverse events [53]. In the subsequent SLS-II, patients who received MMF for 2 years had a similar improvement in FVC to those receiving oral CYC for 1 year, followed by placebo for 1 year, with fewer adverse events [6].

TCZ has been introduced into routine practice following the publication of the phase III results (focuSSced trial [9]) and was approved by the Food and Drug Administration (FDA) for the treatment of SSc-ILD. This study included patients with dcSSc with less than 5 years of duration of the disease and elevated serum markers of inflammation. The mean difference in percent predicted change in FVC from baseline was 167 mL in favor of the TCZ group compared to the placebo group. Subsequent post-hoc analyses [54] showed that SSc-ILD patients enrolled in this study who received TCZ maintained a percent predicted FVC over 48 weeks vs. placebo (−0.1% vs. −6.3%). This was independent of ILD extent on HRCT but was especially marked in severe SSc-ILD patients (those with an extent of >20% on HRCT at baseline). It has been suggested that early treatment may provide a window of opportunity to prevent progression and decline in PFT in this specific population of inflammatory SSc-ILD [55].

The results of the RECITAL study, a RCT comparing the efficacy of RTX with CYC as a first-line treatment for CTD-ILD, including 40% of SSc-ILD cases, have recently been published. In this study, RTX was not superior to CYC, as both drugs had comparable improvements in percent predicted FVC and patient-reported outcomes. However, RTX-treated patients had fewer side effects than CYC-treated patients and were less exposed to glucocorticoids [8]. Based on this RCT, treatment with RTX, alone or in combination with nintedanib and/or MMF, could be considered in some patients with SSc-ILD.

In the area of antifibrotic therapy, nintedanib was approved to slow the decline in PFT in SSc–ILD and chronic FP-ILD patients following the publication of two large phase III RCTs. The SENSCIS study included 576 patients with SSc-ILD and disease duration of less than 7 years, FVC equal to or greater than 40% predicted, and fibrotic ILD of 10% or greater degree on HRCT, of whom 279 (48%) used MMF at baseline [7]. Nintedanib reduced the rate of decline in FVC (ml/year) by 44% over 52 weeks compared to placebo [7]. The INBUILD study enrolled 663 patients with non-IPF chronic PF ILD who had experienced progression within the previous 2 years despite standard of care. Nintedanib slowed in this trial the rate of decline in FVC (mL/year) by 57% vs. placebo over 52 weeks [24]. The most frequent adverse event reported by patients in the SENSCIS and INBUILD studies was diarrhea, which was generally mild and easily managed with temporary or permanent dose reductions or, in the most severe cases, with anti-diarrheal medications [7,24].

Phase II data also suggest that pirfenidone may have some efficacy in other fibrosing ILDs with a PF phenotype, adding to its known efficacy in IPF [28]. Results are pending from a phase II study investigating the effect of pirfenidone combined with MMF in SSc-ILD patients (NCT03221257).

If SSc-ILD worsens despite maximal pharmacological and non-pharmacological efforts, HSCT should be considered in a selected subset of patients [56]. Three RCTs have shown the superiority of HSCT in improving FVC as compared with CYC. There is some data to suggest that treatment with HSCT may even reduce the amount of fibrosis seen on HRCT [56,57]. In order to limit the risks associated with this therapeutic approach, only patients with dcSSc with rapid disease progression and severe visceral involvement should be considered candidates for HSCT, which must always be performed in a referral center.

As a last option, for end-stage pulmonary fibrosis that progresses despite all therapeutic efforts, lung transplantation is the only treatment that can improve long-term outcomes. Results from published studies suggest that survival after lung transplantation in SSc is similar to that of patients with other reasons for lung transplantation [58].

Treatment alternatives should be stratified according to the severity or activity of extrapulmonary manifestations of SSc, the presence of risk factors for ILD progression, overall health status, and patient preferences. Current immunomodulatory and antifibrotic therapies attenuate the consequences of SSc-ILD but have yet to demonstrate a sustained benefit to patient survival, functionality, and quality of life. Questions regarding the preference for upfront or sequential combined immunosuppressive and antifibrotic therapy or the addition of biologics, as is common in other rheumatic diseases, remain areas for further research. In this regard, several studies have reported that combining MMF with nintedanib provides the best scenario for preserving lung function in patients who show disease progression while on a single immunomodulatory agent [3,18,59,60,61]. The optimal therapeutic strategy for managing SSc-ILD patients, including those with a PF phenotype, remains to be determined, particularly given the heterogeneity of the disease. 

## 6. Future Research Agenda

There are many unanswered questions on the research agenda for PF-ILD in SSc: What are the major risk factors associated with the development of this complication in SSc? Is there a biomarker or combination of biomarkers that can be used to detect PF-ILD early? What is the best way to screen for PF-ILD in SSc? What is the natural history of this complication in SSc patients? Will all the patients have a similar course and outcome once PF-ILD has developed? What is the best scheme for monitoring the progression of PF-ILD in SSc patients? How does this complication affect mortality? What is the best therapeutic strategy for PF-ILD in SSc? Will all SSc patients with PF-ILD respond similarly to the same or different therapies?

Given the emerging new concept of PF-ILD, which carries a poor prognosis, effective treatment development will require early detection. In this regard, the identification of biomarkers or risk factors would help to stratify the risk for this complication in each individual SSc patient. Combinations of biomarkers with genetic, demographic, clinical, and imaging findings should improve the diagnosis, monitoring, and management of PF-ILD in SSc patients. New and innovative drugs or drug combinations targeting both the fibrotic and immunologic aspects of the disease have been developed in recent years and will certainly help to improve the management of this serious complication.

## 7. Conclusions

The clinical course of SSc-ILD is highly variable. Early detection to stratify the risk, monitor progression, and initiate treatment when necessary is critical to improving the management of this potentially fatal complication of SSc. Characteristics associated with an increased risk of progressive fibrosing SSc-ILD are demographics (male sex), SSc-specific features such as diffuse disease and short disease duration, serologic markers (ATA), PFT showing decline in FVC and DLCO, and extent of lung involvement on HRCT. The relatively new concept of PF-ILD has generated active clinical research in recent years and has led to a consensus definition accepted by most SSc experts. This increasing research activity highlights a pitfall in current clinical practice, where pharmacological treatment is often initiated after FVC has declined and lung damage has already occurred. Novel treatment approaches are needed and should be aimed at preventing progression to avoid irreversible organ damage from the onset. The importance of using antifibrotic therapy early in PPF, after initial treatment has failed to prevent progression of the disease, is increasingly supported by clinical evidence.

## Figures and Tables

**Table 1:** Criteria for the definition of PPF in two clinical trials and ATS/ERS/JRS/ATAT guidelines.

Diagnostic Criteria	Definition of Progression			Time Period Progression is Assessed
	Lung Function	Symptoms	Chest CT	
Pirfenidone in progressive non-IPF ILD (RELIEF trial) [28]	FVC ≥ 5% decline (absolute)			Within up to 24 months
Nintedanib in progressive non-IPF ILD (INBUILD trial) [24]	FVC ≥ 10% decline (relative); or FVC 5–10% decline and worsening of respiratory symptoms or increased extent of fibrosis on HRCT; or worsening of respiratory symptoms and increased extent of fibrosis on HRCT			Within 24 months
ATS/ERS/JRS/ALAT clinical practice guidelines [23]	At least two of the following three criteria occurred within the past year: (1) Either an absolute decline in FVC > 5% is predicted or an absolute decline in DLCO > 10% is predicted	(2) worsening respiratory symptoms	(3) Radiological evidence of disease progression (one or more of the following): Increased extent or severity of traction bronchiectasis and bronchiolectasis New ground-glass opacity with traction bronchiectasis New fine reticulation Increased extent or increased coarseness of reticular abnormality New or increased honeycombing Increased lobar volume loss	Within 1 year of follow-up

PPF: progressive pulmonary fibrosis; ATS: American Thoracic Society; ERS: European Respiratory Society; JRS: Japanese Respiratory Society; ALAT: Asociacion Latinoamericana de Torax; CT: computed tomography; IPF: idiopathic pulmonary fibrosis; ILD: interstitial lung disease; FVC: forced vital capacity; HRCT: high-resolution computed tomography; DLCO: diffusing capacity of carbon monoxide.

## Data Availability

Not applicable.
